# Nitric oxide, PKC-ε, and connexin43 are crucial for ischemic preconditioning-induced chemical gap junction uncoupling

**DOI:** 10.18632/oncotarget.12087

**Published:** 2016-09-16

**Authors:** Bing Rong, Fei Xie, Tao Sun, Li Hao, Ming-Jie Lin, Jing-Quan Zhong

**Affiliations:** ^1^ The Key Laboratory of Cardiovascular Remodeling and Function Research, Chinese Ministry of Education and Chinese Ministry of Health, The State and Shandong Province Joint Key Laboratory of Translational Cardiovascular Medicine, Qilu Hospital of Shandong University, Jinan, Shandong, China; ^2^ School of Medicine, Shandong University, Jinan, China

**Keywords:** ischemic preconditioning, nitric oxide, PKC-ε, connexin43, gap junction coupling, Pathology Section

## Abstract

Ischemic preconditioning (IPC) maintains connexin43 (Cx43) phosphorylation and reduces chemical gap junction (GJ) coupling in cardiomyocytes to protect against ischemic damage. However, the signal transduction pathways underlying these effects are not fully understood. Here, we investigated whether nitric oxide (NO) and protein kinase C-ε (PKC-ε) contribute to IPC-induced cardioprotection by maintaining Cx43 phosphorylation and inhibiting chemical GJ coupling. IPC reduced ischemia-induced myocardial infarction and increased cardiomyocyte survival; phosphorylated Cx43, eNOS, and PKC-ε levels; and chemical GJ uncoupling. Administration of the NO donor SNAP mimicked the effects of IPC both *in vivo* and *in vitro*, maintaining Cx43 phosphorylation, promoting chemical GJ uncoupling, and reducing myocardial infarction. Preincubation with the NO synthase inhibitor L-NAME or PKC-ε translocation inhibitory peptide (PKC-ε-TIP) abolished these effects of IPC. Additionally, by inducing NO production, IPC induced translocation of PKC-ε, but not PKC-δ, from the cytosolic to the membrane fraction in primary cardiac myocytes. IPC-induced cardioprotection thus involves increased NO production, PKC-ε translocation, Cx43 phosphorylation, and chemical GJ uncoupling.

## INTRODUCTION

Gap junctions (GJs), which are composed of two hexameric connexins, are specialized membrane domains in adjacent cells that allow direct exchange of molecules smaller than 1kDa (chemical coupling) and fast electrical impulses (electrical coupling). In intercalated disks of ventricular cardiomyocytes, changes in electrical GJ coupling can lead to the development of arrhythmia, while changes in chemical GJ coupling are correlated with increases in chemical transmission and cardiomyocyte survival. Connexin43 (Cx43) is the major constituent of ventricular GJ channels, and Cx43 phosphorylation regulates GJ function, including permeability and selectivity [1, 2]. Chemical GJ coupling enhances cardiomyocyte necrosis after ischemia-reperfusion in the heart, and administration of a gap junction blocker limits infarct size [3]. Ischemic preconditioning (IPC), a brief period of ischemia preceding sustained ischemia, salvages the myocardium from necrosis during sustained ischemia-reperfusion [4, 5] by suppressing chemical coupling of GJs in the ischemic myocardium [6, 7]. Protein kinase C (PKC) plays a crucial role both in IPC-induced cardioprotection [8] and in regulating connexin function [9]. Phosphorylation of Cx43 by PKC-ε is a primary mechanism of IPC-induced reduction in chemical GJ permeability after ischemia [6, 10], but the influence of molecules upstream of PKC-ε requires further investigation. The production of nitric oxide (NO), a crucial mediator of vessel physiology [11], also contributes to IPC-induced cardioprotection both in isolated myocytes [12, 13] and in the rat heart [14]. Besides regulating GJs in vascular tissues [15], NO activates electrical coupling of GJs, which suppresses the development of arrhythmias after acute ischemia and reperfusion *in vivo* [16]. However, whether NO modulates chemical GJ coupling in cardiac myocytes by affecting Cx43 phosphorylation status remains unknown. Additionally, whether NO affects IPC-induced cardioprotection processes by inhibiting chemical GJ coupling *in vivo* requires further investigation. NO induces isoform-selective activation and translocation of PKC-ε, which is implicated in IPC-induced cardioprotection during ischemia [17]. We therefore hypothesized that NO is a pivotal upstream effector of PKC-ε and affects Cx43 phosphorylation, thus inhibiting chemical GJ coupling and contributing to IPC-induced cardioprotection. Here, we investigated whether the IPC-NO-PKC-ε-Cx43 signaling cascade, which is thought to be a primary modulator of chemical GJ uncoupling, is responsible for IPC-induced cardioprotection after ischemia.

## RESULTS

### Molecular mechanisms of IPC-induced cardioprotection *in vivo*

**Figure 1:**
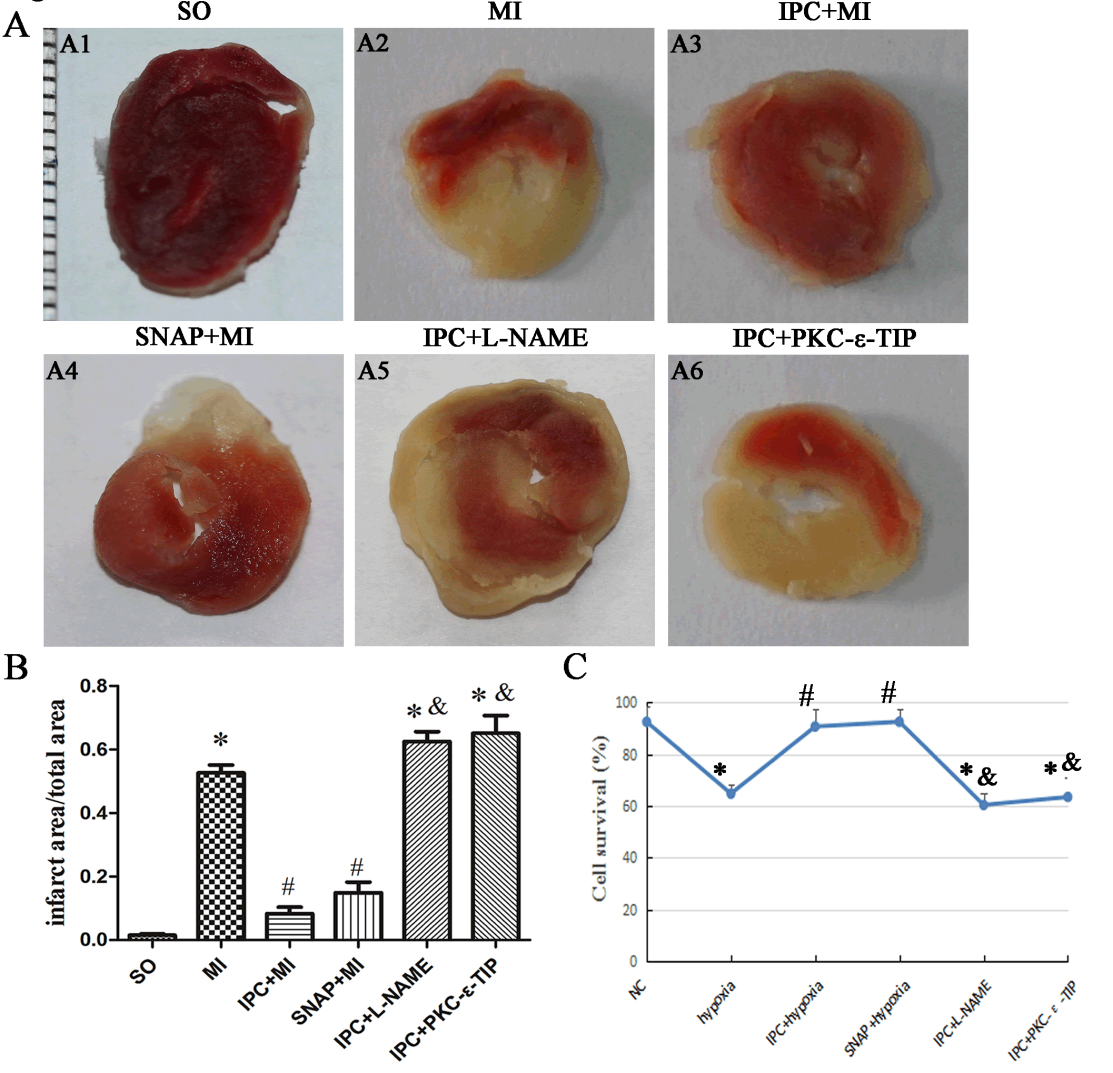
Effects of NO and PKC-ε on IPC-induced cardioprotection *in vivo* and *in vitro* **A**. TTC staining was used to assess myocardium infarct area after different interventions. Red areas represent non-ischemic rat heart issue; white areas represent ischemic rat heart issue. A1-6: sham-operated (SO) group; myocardial ischemia (MI) group; ischemic preconditioning (IPC+MI) group; SNAP (1 nM; 10 min) pretreatment (SNAP+MI) group; L-NAME (1 nM; 20 min) pretreatment (IPC+L-NAME) group; PKC-ε-TIP (100 nM; 30 min) pretreatment (IPC+ PKC-ε-TIP) group (minimum interval of ruler: 1 mm). **B**. Quantitative analysis of infarct area. **C**. CKK-8 was used to evaluate cell survival (line graph). Data are shown as mean ± SEM. *n* = 8 per group. *p < 0.05 *vs*. SO; ^#^*p* < 0.05 *vs*. MI; ^&^ *p* < 0.05 *vs*. IPC.

**Figure 2:**
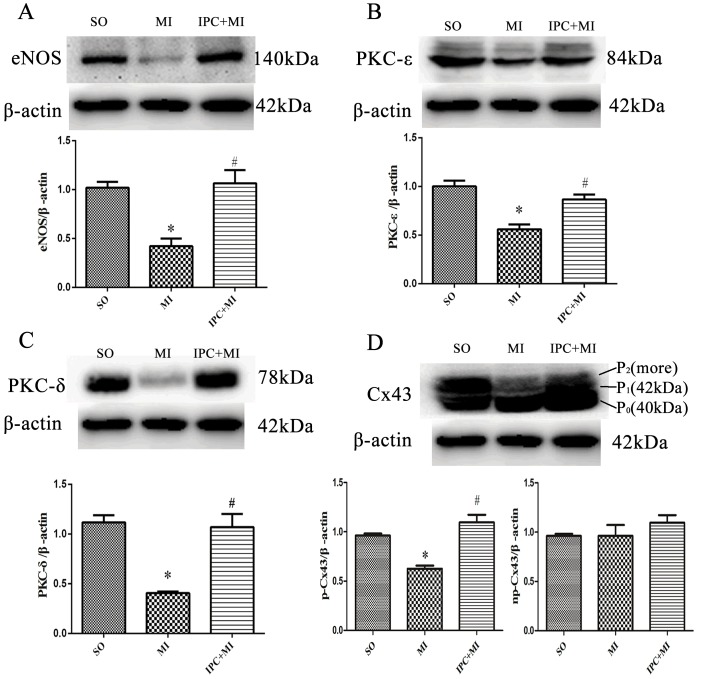
Molecular mechanisms of IPC-induced cardioprotection *in vivo* Western blot assays with anti-eNOS, anti-PKC-δ, anti-PKC-ε, and anti-Cx43 antibodies were performed to measure ventricular tissue protein levels. Representative immunoblots: **A**., **B**., **C**., and **D**. Data are shown as mean ± SEM. *n* = 8 per group. *p < 0.05 *vs*. SO; ^#^*p* < 0.05 *vs*. MI.

In myocardium tissue, ischemia enlarged infarct sizes in heart issues compared to the SO group, and IPC reduced ischemia-induced infarct sizes (Figure 1, A1-3 and 1B). In various cell types, Cx43 mainly manifests as three strong bands after SDS gel electrophoresis reflecting different phosphorylation states. The fastest-migrating band at the P_0_ position corresponds to the non-phosphorylated state, while the two slower-migrating bands, termed Cx43-P_1_ and Cx43-P_2_, correspond to two phosphorylated states. *In vivo*, ischemia decreased, while IPC during ischemia tended to increase, levels of eNOS and PKC-ε and -δ (Figure 2A, 2B and 2C). Additionally, Cx43 phosphorylation decreased after ischemia without IPC compared to the SO group, and IPC reversed this decrease (Figure 2D). These data confirm previous results indicating that NO, PKC-ε, PKC-δ, and phosphorylated Cx43 are involved in IPC-induced cardioprotection [13, 14].

### NO participated in IPC-induced cardioprotection *in vitro*

**Figure 3:**
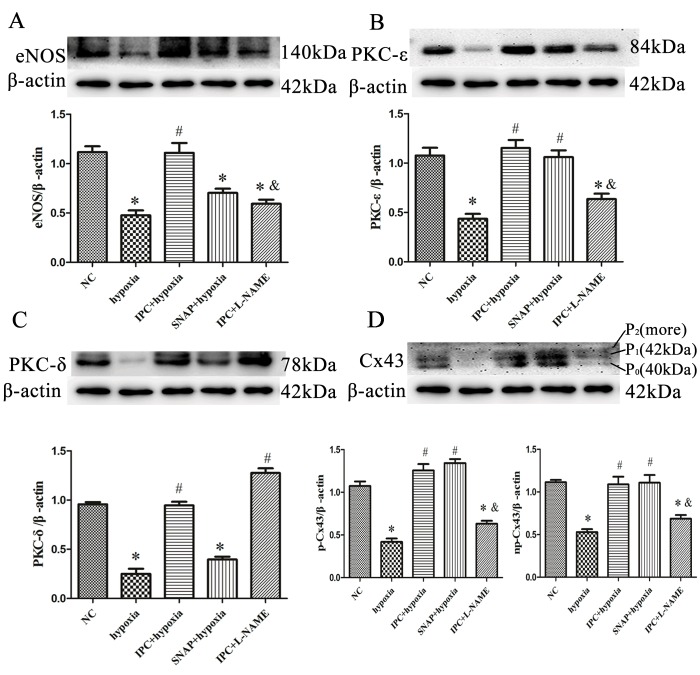
NO participated in IPC-induced cardioprotection *in vitro* Cells were treated with L-NAME (1 nM; 20 min) or SNAP (1 nM; 10 min) prior to IPC or hypoxia treatment, respectively. Representative immunoblots with anti-eNOS **A**., anti-PKC-ε **B**., anti-PKC-δ **C**., and anti-Cx43 **D**. antibodies are shown. Data are shown as mean ± SEM. *n* = 3 per group. *p < 0.05 *vs*. NC; ^#^*p* < 0.05 *vs*. hypoxia; ^&^*p* < 0.05 *vs*. IPC.

To confirm that NO production is essential for IPC-induced cardioprotection, we exposed primary cardiac myocytes to the NO synthase inhibitor L-NAME or the NO donor SNAP. *In vitro*, IPC reversed the hypoxia-induced decrease in cell survival (Figure 1C). SNAP increased cell survival similarly to IPC, while L-NAME abolished the IPC-induced increase in cell survival (Figure 1C). Preincubating cells with L-NAME before IPC abolished most IPC-induced effects, reducing eNOS, PKC-ε, and phosphorylated Cx43 levels (Figure 3A, 3B and 3D); L-NAME did not decrease IPC-induced PKC-δ levels (Figure 3C). The effects of SNAP administration before hypoxia were similar to those of IPC; Cx43 band patterns gradually returned to IPC-induced patterns and PKC-ε levels increased (Figure 3B and 3D). SNAP did not affect the hypoxia-induced decrease in PKC-δ levels (Figure 3C). Additionally, SNAP, which provides NO directly, did not affect eNOS levels, which decreased after hypoxia (Figure 3A). Overall, NO production activated PKC-ε and maintained phosphorylated Cx43 levels, which play important roles in IPC-induced cardioprotection.

### Treatments altered PKC-δ and PKC-ε distribution *in vitro*

**Figure 4:**
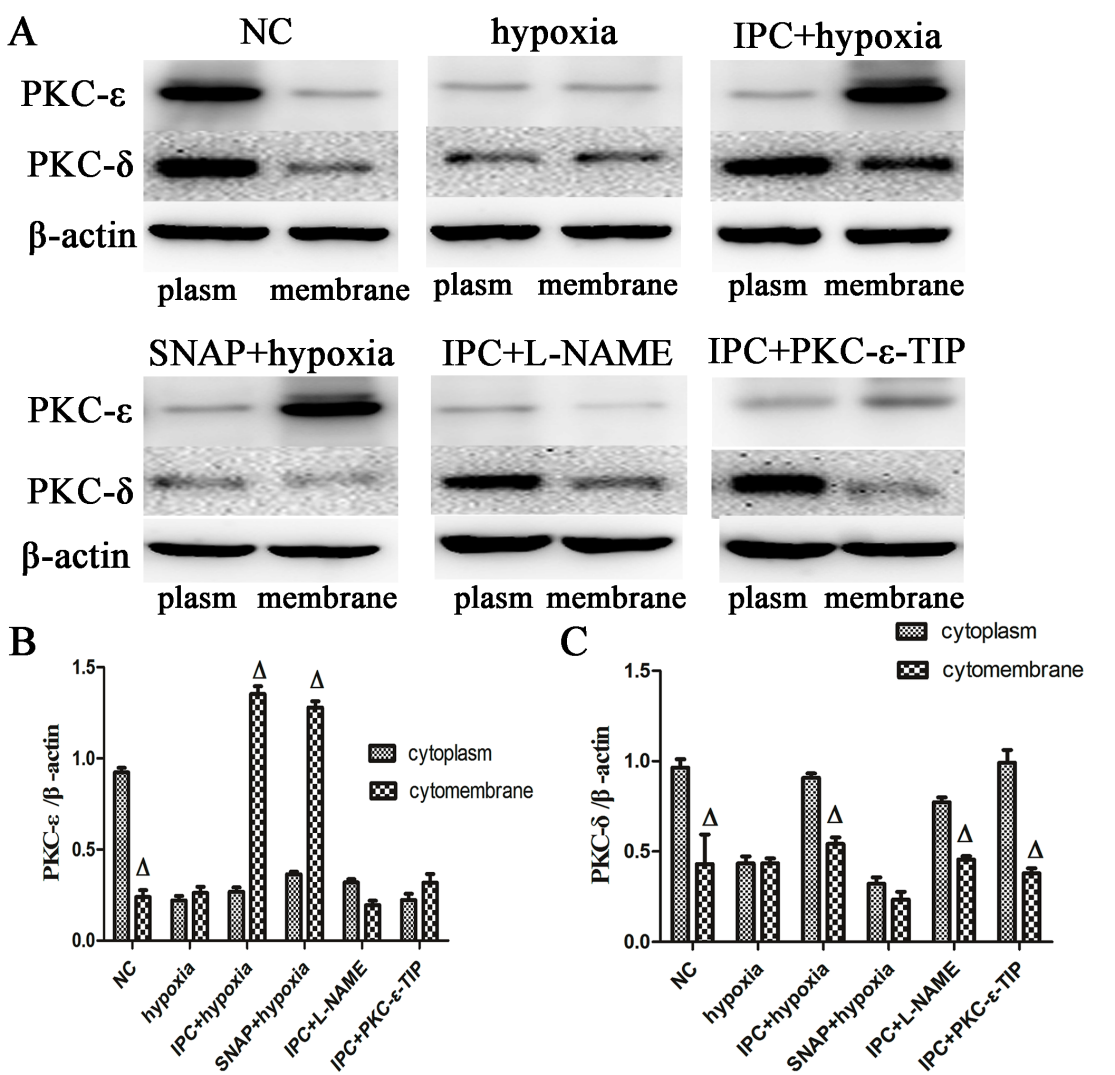
Altered PKC-δ and PKC-ε distribution after different interventions in vitro Membrane and cytoplasmic proteins were separated from cells in the different treatment groups. Western blot assays were performed with anti-PKC-δ and anti-PKC-ε antibodies (representative immunoblots: **A**.,. **B**. Quantitative analysis of PKC-ε protein. **C**. Quantitative analysis of PKC-δ protein. Data are shown as mean ± SEM. *n* = 3 per group. ^Δ^*p* < 0.05 *vs*. cytoplasm proteins.

Immunoblotting was used to measure the relative proportions of PKC-ε and PKC-δ in cytosolic and membrane fractions from primary cardiac myocytes. In the NC group, PKC-ε and PKC-δ content was higher in cytosolic fractions than in membrane fractions. Hypoxia induced marked decreases in both PKC-ε and PKC-δ levels (Figure 4A, 4B, and 4C). IPC induced the translocation of PKC-ε from cytosolic to membrane fractions (Figure 4A and 4B); PKC-δ distribution in the membrane and cytoplasm after IPC was similar to that of the NC group (Figure 4A and 4C). Both SNAP and IPC tended to induce the translocation of PKC-ε, and L-NAME abolished the IPC-induced translocation of PKC-ε (Figure 4A and 4B). Additionally, neither SNAP nor L-NAME affected hypoxia- or IPC-induced changes in PKC-δ levels and locations (Figure 4A and 4C). In conclusion, NO induced the translocation of PKC-ε, but not PKC-δ, from cytosolic to membrane fractions after IPC. We therefore used PKC-ε-TIP to confirm the IPC-induced translocation of PKC-ε. As shown in Figure 4A and 4C, PKC-ε-TIP prevented IPC-induced translocation of PKC-ε. Thus, NO participated in IPC-induced cardioprotection by increasing the translocation of PKC-ε, but not PKC-δ, from the cytosol to the membrane.

### PKC-ε was involved in IPC-induced cardioprotection *in vitro*

**Figure 5:**
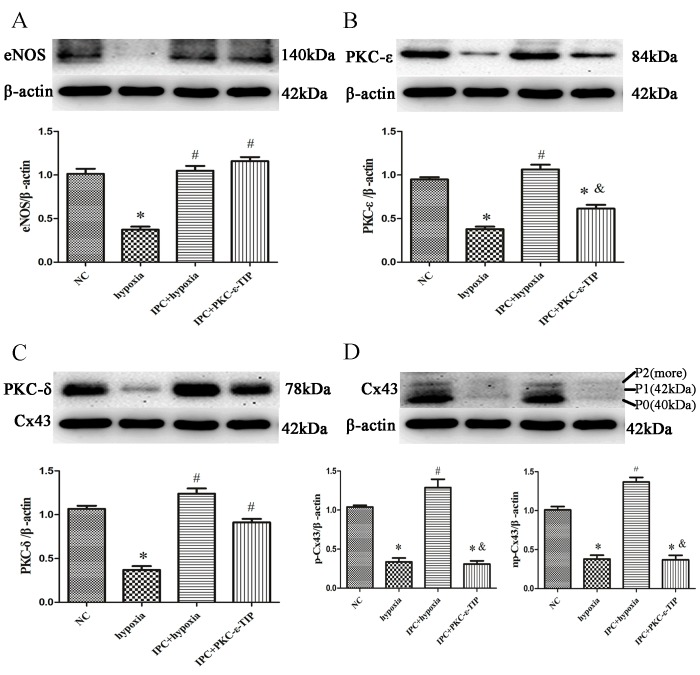
PKC-ε was involved in IPC-induced cardioprotection in vitro Cells were treated with PKC-ε-TIP (100 nM; 30 min) prior to IPC treatment, and protein samples were extracted. Representative immunoblots with anti-eNOS **A**., anti-PKC-ε **B**., anti-PKC-δ **C**., and anti-Cx43 **D**. antibodies are shown. Data are shown as mean ± SEM. *n* = 3 per group. *p < 0.05 *vs*. NC; ^#^*p* < 0.05 *vs*. hypoxia; ^&^*p* < 0.05 *vs*. IPC.

Pre-treatment with PKC-ε-TIP attenuated the IPC-induced increase in primary cardiac myocyte survival (Figure 1C). PKC-ε-TIP did not affect IPC-induced increases in eNOS levels, reduced PKC-ε levels, and had no impact on PKC-δ levels (Figure 5A, 5B and 5C). Additionally, PKC-ε-TIP inhibited IPC-induced Cx43 phosphorylation (Figure 5D). Overall, PKC-ε, a downstream molecule of NO, plays a major role in IPC-induced Cx43 phosphorylation maintenance and cardioprotection.

### Effects NO and PKC-ε on IPC-induced cardioprotection *in vivo*

**Figure 6:**
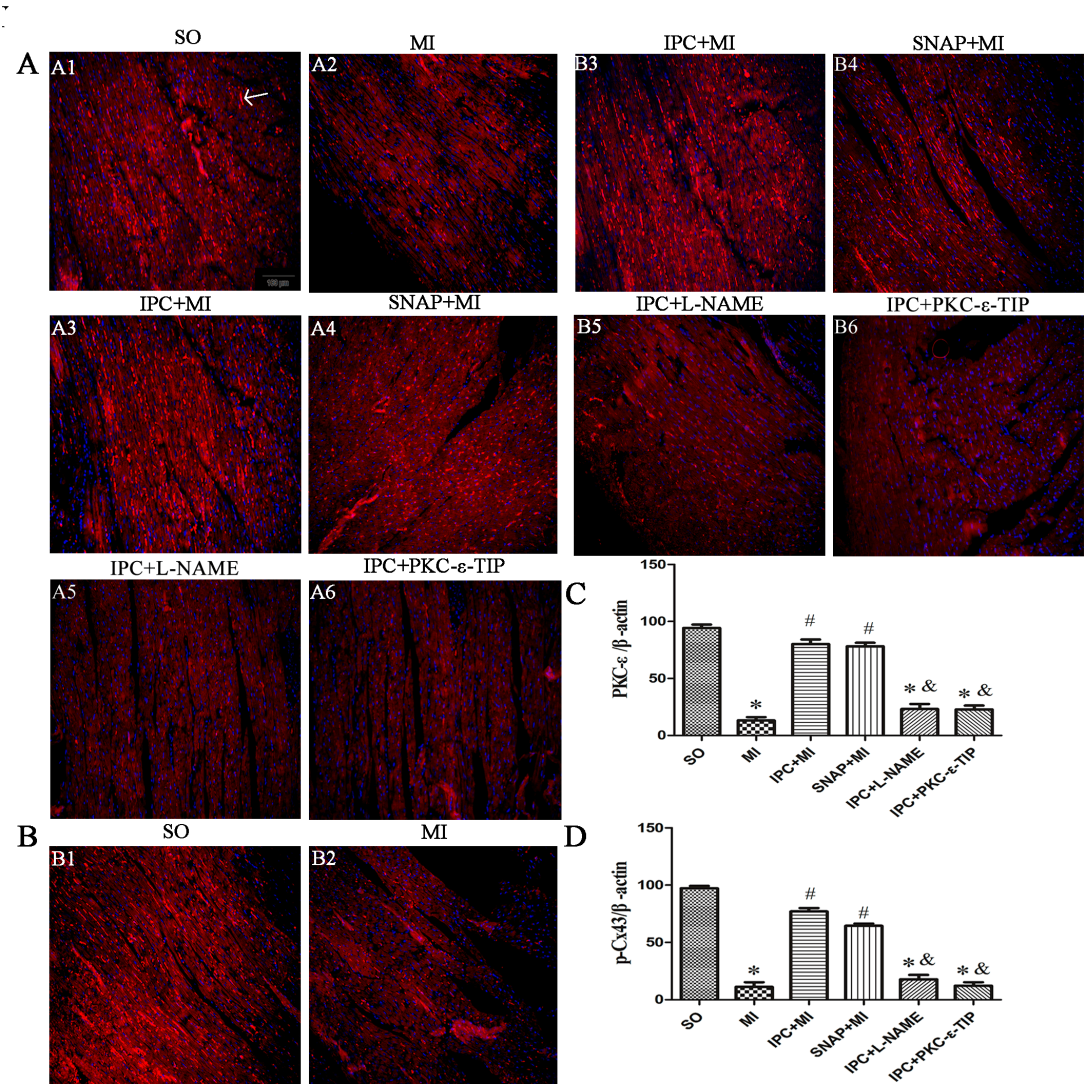
Effects NO and PKC-ε on IPC-induced cardioprotection *in vivo* Immunofluorescence staining was performed to measure PKC-ε and phosphorylated Cx43 levels. PKC-ε or phosphorylated Cx43 protein (arrow) are stained red; nuclei are stained blue (DAPI staining) (scale bar, 100 μm). **A**. Changes in PKC-ε levels after different interventions. **B**. Changes in phosphorylated Cx43 levels after different interventions. **C**. Quantitative analysis of PKC-ε protein levels. **D**. Quantitative analysis of phosphorylated Cx43 protein levels. Data are shown as mean ± SEM. *n* = 8 per group. *p < 0.05 *vs*. SO; ^#^*p* < 0.05 *vs*. MI; ^&^*p* < 0.05 *vs*. IPC.

In myocardium tissue, SNAP reduced infarct size, and L-NAME abrogated IPC-induced reductions in infarct size (Figure 1, A4-5 and B). PKC-ε-TIP also counteracted the effects of IPC and increased infarct size (Figure 1, A6 and B). *In vivo*, SNAP increased PKC-ε (Figure 6, A4 and C) and phosphorylated Cx43 (Figure 6, B4 and D) levels similarly to IPC (Figure 6, A3, B3, C and D, respectively); L-NAME prevented IPC-induced increases in PKC-ε and phosphorylated Cx43 levels (Figure 6, A5, B5, 6C and 6D). PKC-ε-TIP administration also blocked the IPC-induced increase in phosphorylated Cx43 levels (Figure 6B6 and D), but did not affect eNOS increase in phosphorylated Cx43 levels (Figure 6, B6 and 6D), but did not affect eNOS levels (not shown). These data suggest that NO activates the downstream molecule PKC-ε, which then phosphorylates Cx43, during IPC-induced cardioprotection.

### NO and PKC-ε promoted chemical GJ uncoupling during IPC treatment *in vitro* and *in vivo*

**Figure 7:**
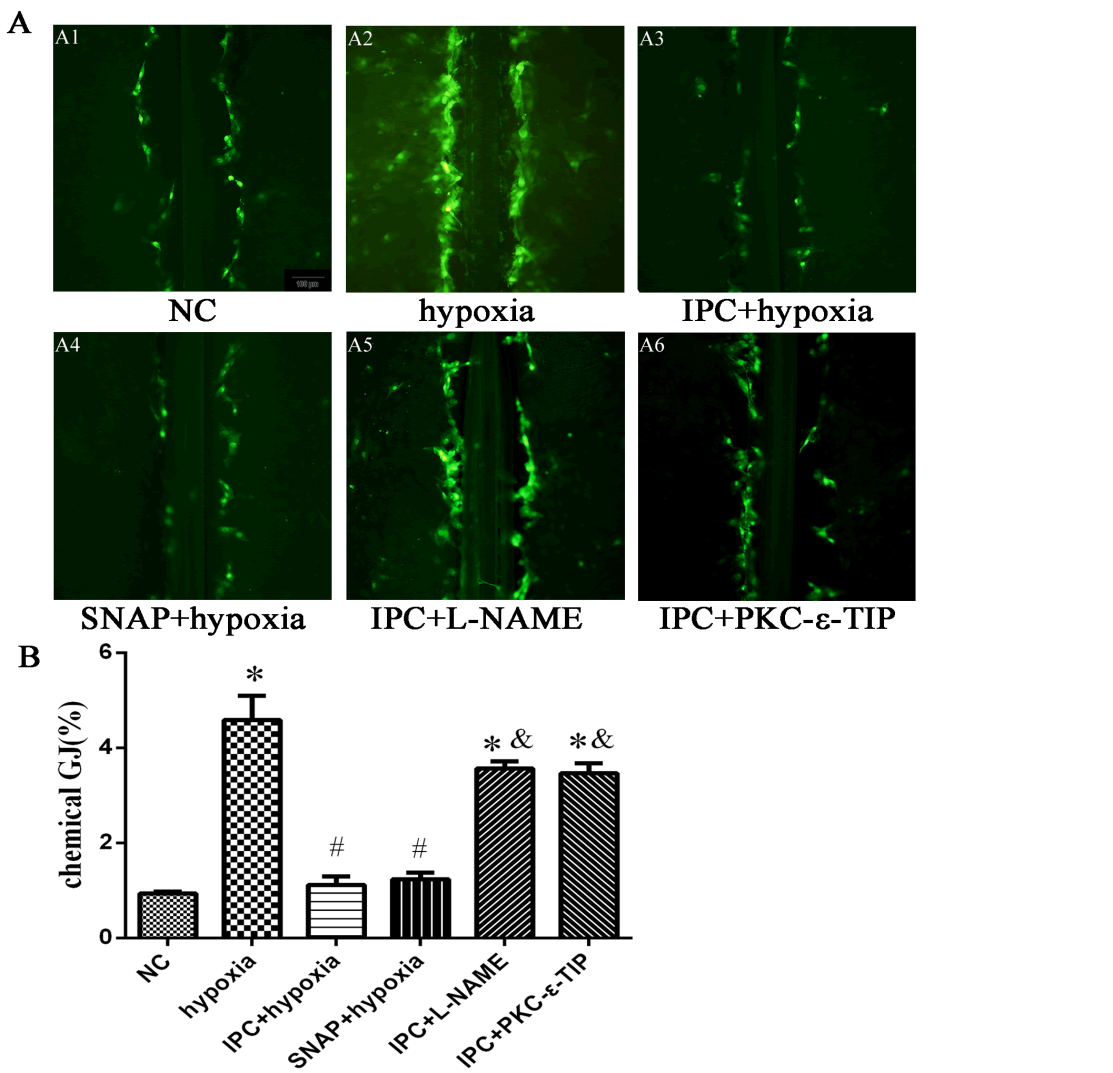
NO and PKC-ε promoted chemical GJ uncoupling during IPC *in vitro* **A**. Chemical GJ permeability was measured by scrape loading after cells were exposed to different treatments (scale bar, 100 μm). **B**. Quantitative analysis of chemical GJ coupling. Data are shown as mean ± SEM. *n* = 3 per group. *p < 0.05 *vs*. NC; ^#^*p* < 0.05 *vs*. hypoxia; ^&^*p* < 0.05 *vs*. IPC.

**Figure 8:**
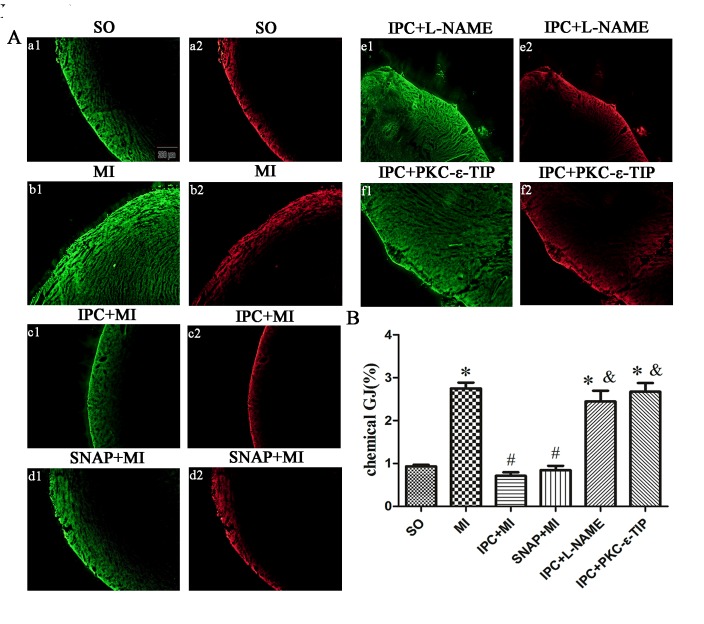
NO and PKC-ε promoted chemical GJ uncoupling during IPC *in vivo* Areas of LY staining (yellow) indicate chemical GJ permeability; areas of RD staining (red) indicate sarcolemma disruption. Areas stained with RD were subtracted from areas stained with LY to evaluate chemical GJ coupling. **A**: representative images after different interventions (scale bar, 200 μm). **B**: Quantitative analysis of chemical GJ coupling. Data are shown as mean ± SEM. *n* = 8 per group. *p < 0.05 *vs*. SO; ^#^*p* < 0.05 *vs*. ischemia; ^&^*p* < 0.05 *vs*. IPC.

In primary cardiac myocytes, hypoxia-induced chemical GJ coupling increased to 4.9 times the NC level (Figure 7, A1-2 and B), and IPC suppressed chemical GJ coupling by 76% compared to the hypoxia group (Figure 7, A3 and B). SNAP decreased chemical GJ coupling similarly to IPC (Figure 7, A4 and B), while pre-incubation with L-NAME abolished the IPC-induced decrease in chemical GJ coupling (Figure 7, A5 and B). As shown in Figure 7A6 and B, PKC-ε-TIP also abolished the IPC-induced decrease in chemical GJ coupling. In conclusion, IPC causes chemical uncoupling of GJs via NO and PKC-ε *in vitro*. In myocardium tissue, ischemia increased chemical GJ coupling (Figure 8, b1 and b2), while IPC attenuated this effect (Figure 8, c1 and c2). SNAP administration decreased chemical GJ permeability similarly to IPC (Figure 8, d1 and d2), and L-NAME counteracted the effects of IPC on chemical GJ coupling (Figure 8, e1 and e2). Additionally, PKC-ε-TIP also abolished the IPC-induced decrease in chemical GJ permeability (Figure 8, f1 and f2). These data indicate that NO and PKC-ε are vital mediators of IPC-induced chemical GJ uncoupling *in vivo*.

## DISCUSSION

IPC exerts cardioprotective effects against ischemia-reperfusion injury [18] by reducing chemical GJ coupling [7], and GJ inhibitor administration during ischemia and reperfusion reduces infarct sizes [19]. In the present study, IPC decreased myocardial infarct sizes and primary cardiac myocyte survival after ischemia. Additionally, the inhibitory effect of IPC on chemical GJ coupling was confirmed using the Lucifer yellow tracer in primary cardiac myocytes and myocardium tissues. IPC induces chemical GJ uncoupling through various molecular mechanisms in ischemic myocardium [6], but the main molecular cascade underlying IPC-induced chemical GJ uncoupling is unknown. Phosphorylation of Cx43 is an important regulator of GJ communication [1,2]. Cx43 is activated by many protein kinases, including PKC-ε, Src tyrosine kinase, and p38mitogen-activated protein kinase (p38MAPK), which participate in IPC-induced uncoupling of chemical GJs [10]. In our study, Cx43 phosphorylation levels decreased after ischemia. However, IPC maintained Cx43 phosphorylation, likely by increasing the co-localization of protein kinases (such as PKC-α, PKC-ε, and p38MAPK) with Cx43, during sustained ischemia *in vivo* in pig hearts [20]. We found that, in addition to the maintenance of Cx43 phosphorylation, chemical GJ uncoupling is essential for IPC-induced cardioprotection. Levels of non-phosphorylated Cx43 did not change obviously in rat hearts, but decreased in primary cardiac myocytes, after ischemia. Increased proteasome degradation of non-phosphorylated Cx43 in primary cardiac myocytes compared to rat hearts after ischemia may account for this result [21].

NO, a key signaling molecule induced by IPC, plays a role in cardioprotection against ischemia-reperfusion injury [22, 23]. Several studies strongly suggest that NO originating from vascular endothelium contributes to IPC-induced cardioprotection during ischemia [14]. Additionally, NO contributes to IPC-induced cardioprotection in isolated myocytes, suggesting that myocytes are responsible for NO production during IPC [12, 13]. In our study, NO contributed to IPC-induced cardioprotection, which was characterized by reduced infarct areas and increased cell survival. There are studies suggesting that NO modulates GJ permeability and the expression of Cx isoforms in non-cardiac tissues [24, 25]. Additionally, NO may affect electrical GJ coupling not only in the vasculature [15], but also in cardiac myocytes [16]; whether NO is involved in IPC-induced chemical GJ uncoupling in cardiac myocytes remains unknown. Here, using L-NAME and SNAP, we demonstrated that NO-mediated pathways reduced chemical GJ coupling in cardiac myocytes.

PKC is also involved in IPC-induced protection against ischemia-reperfusion injury [26, 27]. Among PKC isoforms, the ε- and δ-isoforms are the most important for IPC-induced effects; the PKC-ε isoform specifically plays a critical role in IPC-induced reductions in infarct size [28]. In the present study, IPC increased PKC-ε protein levels, and results obtained after PKC-ε-TIP administration indicated that PKC-ε was involved in IPC-induced cardioprotection. Non-selective inhibition of PKC by chelerythrine or calphostin C abrogates IPC-induced delayed uncoupling and Cx43 redistribution, suggesting that PKC is required for the effects of IPC on GJ coupling [29]. The effects of administration of PKC-ε-TIP and a PKC activator, phorbol 12-myristate 13-acetate, suggest that phosphorylation of Cx43 at Ser368 by PKC-ε is the primary mechanism by which IPC suppresses chemical GJ coupling [10, 30]. Both IPC-induced translocation of PKC-ε to GJs and increases in the co-immunoprecipitation of PKC-ε with connexin-43 (Cx43) confirm the importance of PKC-ε in this regard [10, 31]. Our results indicate that IPC induced the translocation and activation of PKC-ε, which suppressed chemical GJ coupling via Cx43 phosphorylation.

Many studies have confirmed that PKC activation contributes to the effects of IPC through various molecular mechanisms, including mK_ATP_ channels [32] and NO [13]. PKC-ε isoform activation and translocation play pivotal roles both in NO donor-induced IPC (exogenous NO) and ischemia-induced IPC (endogenous NO) [17]. We found that NO promoted PKC-ε isoform activation and translocation, which mimicked the effects of IPC. Meanwhile, L-NAME, SNAP, and PKC-ε-TIP administration altered PKC-ε isoform content and location in a manner consistent with changes in Cx43 phosphorylation status and chemical GJ uncoupling in both primary cardiac myocytes and myocardium tissue. Thus, IPC-induced NO generation inhibited chemical GJ coupling by activating PKC-ε translocation and maintaining Cx43 phosphorylation in cardiac myocytes.

The δ-Opioid receptor plays a vital role in IPC-induced cardioprotection via the PKC-δ isoform. Direct phosphorylation of Cx43 Ser368 mediated by PKC-ε, but not PKC-δ, was required for δ-Opioid receptor-induced reduction in GJ permeability in ischemic myocardium [33]. Here, IPC decreased PKC-δ content, but PKC-δ levels did not affect NO-induced Cx43 phosphorylation. IPC also promoted the translocation of PKC-ε, but not PKC-δ, from cytosolic to membrane fractions, and NO had no effect on translocation of PKC-δ. These data suggest that NO contributed to IPC-induced chemical GJ uncoupling by stimulating downstream PKC-ε rather than PKC-δ. However, PKC-δ might mediate IPC-induced cardioprotection through other molecular mechanisms.

In conclusion, IPC-induced NO production contributed to cardioprotection after ischemia by promoting chemical GJ uncoupling via the activation and translocation of PKC-ε, which phosphorylated Cx43.

## MATERIALS AND METHODS

### Ethics statement

The experimental protocol complied with the Animal Management Rules of the Chinese Ministry of Health (Document No. 55, 2001) and was approved by the Animal Care and Use Committee of Shandong University. Adult (about 8 weeks; 200-250 g) and neonatal (1-3 days; primary cardiac myocytes) Wistar rats were purchased from the animal organization of the Medical School at Shandong University (Shandong, China). All animals were maintained at the Key Laboratory of Cardiovascular Remodeling and Function Research in Qilu Hospital of Shandong

University, fed an ordinary diet and filtered tap water, and housed at 20-24°C with a relative humidity of 40-60%. All animal procedures were performed according to ARRIVE guidelines. Adult and neonatal rats were euthanized.

### Reagents and antibodies

N^G^-nitro-L-arginine methyl ester (L-NAME; eNOS inhibitor) was purchased from Beyotime (China). S-nitroso-N-acetylpenicillamine (SNAP; NO donor) and PKC-ε translocation inhibitory peptide (PKC-ε-TIP; inhibitor of PKC-ε translocation and activation) were obtained from Calbiochem/EMD Biosciences (Germany). Anti-PKC-ε antibody was obtained from Abcam (NK). Lucifer yellow (LY), rhodamine-conjugated dextran (RD), TTC dye, and cell counting kit-8 (CCK-8) were obtained from Sigma (USA). Anti-PKC-δ and anti-eNOS antibodies were obtained from Cell Signaling Technology (CST, USA). Cx43 polyclonal antibody directed against a segment of the 3rd cytoplasmic domain (C-terminal portion) of rat Cx43 and recognizing non-phosphorylated (np-Cx43) and phosphorylated (p-Cx43) forms of Cx43 was purchased from Invitrogen.

### Isolated perfused heart preparation and study groups

After induction of anesthesia with sodium pentobarbital (40 mg/kg; intraperitoneal injection), rats were ventilated artificially with a volume-controlled rodent respirator at 70 strokes/min. Hearts were rapidly excised, mounted on a Langendorff apparatus, and retrogradely perfused with modified Krebs-Henseleit (K-H) solution (127 mM NaCl; 17.7 mM NaHCO_3_; 5.1 mM KCl; 1.5 mM CaCl_2_; 1.26 mM MgCl_2_; 11 mM D-glucose; pH 7.4). The solution was saturated with 95% O_2_ and 5% CO_2_ at constant pressure (75 mmHg) and temperature (37°C).

Seventy-two adult male Wistar rats (200-250 g) were randomly assigned to six groups receiving the following treatments: sham-operated (SO, n=12) group: hearts were perfused persistently for 30 min; myocardial ischemia (MI, n=12) group: hearts were subjected to 30 min of zero-flow global ischemia; ischemic preconditioning (IPC+MI, n=12) group: hearts were preconditioned by zero-flow (5 min) and reperfusion (10 min) periods 3 times before 30 min global ischemia; SNAP pretreatment (SNAP+MI, n=12) group: K-H solution containing SNAP (1 nM) was pre-administered to hearts for 10 min before 30 min of zero-flow global ischemia; L-NAME pretreatment (IPC+L-NAME, n=12) group: K-H solution containing L-NAME (1 nM) was pre-administered to hearts for 20 min before IPC; PKC-ε-TIP pretreatment (IPC+PKC-ε-TIP, n=12) group: K-H solution containing PKC-ε-TIP (100 nM) was pre-administered to hearts for 30 min before IPC.

### Triphenyltetrazolium chloride (TTC) staining

Fresh heart tissue (primarily left ventricles) was cut into 2-mm thick tissue blocks which were incubated in a 1% TTC solution at 37°C in the dark. After 20 min of incubation, the slices were immersed in 4% paraformaldehyde. Images of the sections were obtained using an Imaging-Pro-Plus system (Olympus, Silver Spring, MD, USA). The infarct area in each section was measured and is expressed as the ratio of ipsilateral hemisphere infarct area/ipsilateral hemisphere total area.

### Determination of GJ permeability in myocardium

Chemical GJ coupling was examined using LY, an anionic gap junction tracer, and RD. Fresh transmural ventricular tissue was made by cutting with a surgical blade along the long axis, and the tissue was then immersed in phosphate-buffered saline (PBS; 140 mM NaCl, 2.68 mM KCl, 8.1 mM Na_2_HPO_4_, 1.47 mM KH_2_PO_4_, pH 7.4) containing LY (1.5 mg/ml) and RD (3.5 mg/ml) at 37°C. After 15 min, tissue blocks were fixed with 4% paraformaldehyde, frozen, and sectioned (25 μm) for fluorescence microscopy. Areas stained with RD were subtracted from areas stained with LY for evaluation of chemical GJ coupling.

### Immunofluorescence histochemistry of myocardium

Myocardial tissues were fixed in 4% paraformaldehyde, embedded in paraffin, and cut into sections (4 μm). Sections were incubated with anti-PKC-ε antibody (1/2000) and Cx43 polyclonal antibody (1/100) overnight at 4°C with slow shaking. Next, the sections were immunoblotted with goat anti-rabbit IgG-conjugated Rhodamine (TRITC) for 30 min at room temperature. Immunofluorescence images were obtained and analyzed using confocal laser microscopy.

### Cell culture

Primary cardiac myocytes were isolated and cultured as previously described [34]. Cells were grown in Dulbecco's modified Eagle's medium (DMEM) supplemented with 10% fetal bovine serum (FBS; Gibco BRL Life Technologies), 4.5 g/L D-glucose and L-glutamine, and 110 mg/L sodium pyruvate. Cells were trypsinized, plated in 35 mm Petri dishes in the same medium, and incubated in a humidified atmosphere with 95% air/5% CO_2_ at 37°C. For normoxia, cells were incubated in a 37°C incubator with a 95% air/5% CO_2_ atmosphere for 30 min (Negative Control group, NC). For hypoxia, cells were cultured in a 94% N_2_, 5% CO_2_, and 1% O_2_ atmosphere for 30 min (hypoxia simulates ischemia; hypoxia group). For IPC, cells were preconditioned with three cycles of 5 min hypoxia and 10 min recovery before 30 min hypoxia (IPC+hypoxia group).

### Treatment of cells and preparation of samples

To evaluate the effects of NO on cardiac protection, primary cardiac myocytes were stimulated with L-NAME before IPC (IPC+L-NAME group) or SNAP before ischemia (SNAP+hypoxia group). To examine the effects of PKC-ε and −δ isoforms on IPC-induced cardioprotection, cells were treated with PKC-ε translocation inhibitory peptide (PKC-ε-TIP) before IPC (IPC+PKC-ε-TIP group). The reagents were dissolved in PBS or dimethyl sulfoxide (DMSO) and added to triplicate Petri dishes at the concentrations and time -points indicated in figure legends. Protein samples were extracted as previously described [35]. Additionally, cytoplasm and membrane proteins (including PKC-ε, PKC-δ) were extracted using a membrane and cellular plasma protein extraction kit.

### Western blotting

Protein samples were loaded and separated as previously described [35]. Membranes were immunoblotted with specific primary antibodies (anti-PKC-ε, anti-PKC-δ, anti-eNOS, 1:2000) and anti-Cx43 antibody (1:100) overnight at 4°C.

### Determination of GJ function by scrape loading

Primary cardiac myocytes were processed as previously described [35]. Primary cardiac myocytes were grown in 35 mm Petri dishes for 48 h prior to experiments. The confluent cell layer was rinsed twice with PBS, followed by incubation in fluorescent dye (Lucifer Yellow; 0.05 w/v in PBS without Ca^2+^ and Mg^2+^), and the cell monolayer was then cut 5-6 times with a surgical scalpel. After 5 min, incubation was ended by removing the Lucifer Yellow solution. Dishes were then washed four times with PBS and fixed in 4% formaldehyde in PBS. Migration of dye from the scrape line was measured using an inverted epifluorescent microscope (T200, Nikon, Japan). ImageJ was used for analysis. GJ coupling levels were determined by fluorescent dye diffusion distance. Exposure to chlordane (30 μM) for 1 h completely blocked GJ coupling in the cells (data not shown); under these conditions, fluorescent cells that obtained dye directly through the scraping process defined zero GJ coupling.

### Cell counting kit-8 cell survival assay

A cell counting kit-8 (CCK-8) was used to assess cell survival according to the manufacturer's instructions. Primary cardiomyocytes were plated in 96-well plates. After different treatments, CCK-8 solution (10 μL) was added to the cells and the plate was incubated for 2 h at 37°C. Absorbance at 450 nm and reference absorbance at 630 nm were measured using a microplate reader. The cell survival percentage was calculated with the following formula: cell viability (%) = (mean absorbance after treatment)/(mean absorbance of NC) × 100.

### Statistical analysis

Data are reported as mean ± SEM of at least three independent experimental replicates and were compared using one-way ANOVAs in SPSS 18.0 statistical software. Differences were considered significant at *p*<0.05.
